# Silk Suture Hypersensitivity Masquerading as Recurrent Hypoglossal Nerve Stimulator Infections

**DOI:** 10.1002/oto2.70058

**Published:** 2025-02-26

**Authors:** John J. Ceremsak, Basil M. Kahwash, Holly Budnick, David T. Kent

**Affiliations:** ^1^ Department of Otolaryngology–Head and Neck Surgery Vanderbilt University Medical Center Nashville Tennessee USA; ^2^ Division of Allergy, Pulmonary and Critical Care Medicine Vanderbilt University Medical Center Nashville Tennessee USA

**Keywords:** hypersensitivity, hypoglossal nerve stimulation, obstructive sleep apnea

Hypoglossal nerve stimulation (HNS) is a novel surgical technique for the treatment of obstructive sleep apnea (OSA) that requires implantation of three electronic components in the soft tissues of the head and neck. While infection is a significant risk in any procedure involving sterile foreign body implantation, there is little extant literature on hypersensitivity responses resulting in device removal or replacement. Here we document a case of a foreign body hypersensitivity reaction to surgical materials masquerading clinically as recurrent infections of an HNS device. This report was exempt from IRB review.

## Case Report

A 59‐year‐old male with severe OSA underwent right‐sided HNS implantation without complication. Intraoperatively, size 0 silk sutures were used to anchor the implant to the soft tissues of the patient's chest wall. On postoperative Day 13, the patient's right chest wound became erythematous, indurated, and tender. The patient was prescribed trimethoprim‐sulfamethoxazole therapy but experienced worsening symptoms and ultimately dehiscence of the wound, exposing the implant and draining serosanguinous fluid.

The patient subsequently underwent complete explantation of the HNS device for presumed infection with complete recovery. Notably, no frank purulence was encountered intraoperatively. Six weeks later, he underwent repeat implantation on the left side but experienced a nearly identical postoperative clinical course and subsequent dehiscence of the chest wound, necessitating a return to the operating room. Intraoperatively, the chest wound again exhibited only serosanguinous drainage, prompting consideration of alternative etiologies including foreign body hypersensitivity reaction. The patient's recovery was uncomplicated, and the intraoperative chest wound cultures were ultimately negative for bacterial growth.

**Figure 1:**
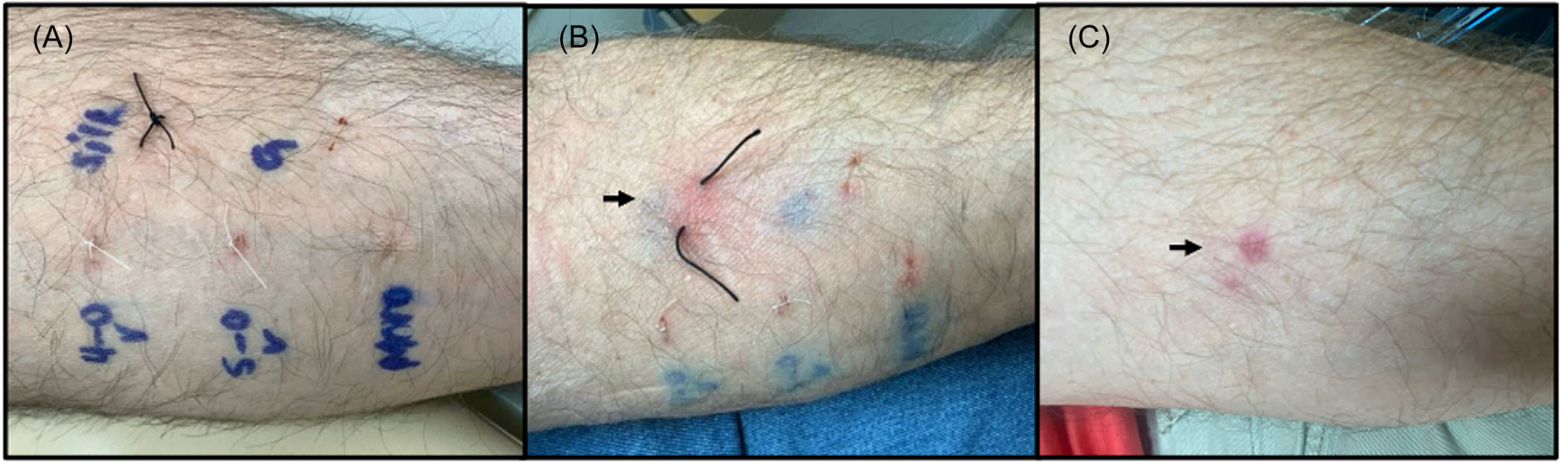
Suture hypersensitivity testing. (A) Clockwise from top‐left: Silk, Fast gut, 4‐0 Monocryl, 5‐0 fast gut, 4‐0 Vicryl. (B) Day 5 silk hypersensitivity (arrow). (C) 3 months postprocedure.

The patient underwent contact dermatitis testing with multiple surgical materials. A thin‐layer cutaneous patch test to surgical materials and metals was negative, but suture material testing demonstrated a hypersensitivity reaction to silk suture (Figure 1).^1^ Based on these findings, the patient underwent repeat implantation 6 weeks later with polyethylene suture used for device anchoring instead of silk. He healed well.

## Discussion

Adverse reactions to implanted materials are uncommon but may be an underrecognized phenomenon often mistakenly attributed to wound infection. In a review of adverse reactions seen in thyroid surgery, Hocwald et al suggested that reactions may be classified based on their time course and severity.^2^ Mild reactions are characterized by erythema of the surgical scar, occurring 7 to 10 days after surgery. More severe reactions are characterized by involvement of deeper structures with small abscesses and granulomas. Our patient developed progressive dehiscence only a few weeks after surgery, suggesting a particularly severe early reaction.

Silk suture has been widely used for centuries and has a known hypersensitivity profile that is generally considered favorable compared to synthetic suture materials. Adverse reactions, however, have been described in a variety of case reports. Eldridge and Wheeler reviewed a series of 526 thyroidectomies and reported suture granuloma formation in 8% of patients receiving silk versus 0% in those without.^3^ Of note, suture complications occurred weeks or months after surgery, and there was an increased rate of granuloma formation in patients with a higher silk burden (9% vs 3%). Suture material hypersensitivity may be particularly challenging to diagnose because reactions may not be observed unless it is implanted across the dermis.^4^

HNS implantation is generally considered a safe procedure. A retrospective review of publicly reported adverse events associated with HNS performed by Bellamkonda et al found that the most common reason for explantation of the HNS device was infection while the most common cause for revision surgery was migration of the IPG.^5^ The authors did note one reported instance of “allergy” to the IPG, however further details were not available, and the reaction did not lead to revision surgery or explantation.

## Conclusion

In this case report, we present a patient who underwent multiple rounds of HNS implantation and explantation for a presumed device infection who was ultimately found to have a silk suture hypersensitivity. Anchoring of the IPG with a non‐silk suture resulted in uncomplicated healing. Surgeons should be aware of surgical material hypersensitivity as a rare cause of postoperative symptoms that may be mistaken for infection in patients undergoing HNS surgery.

## Author Contributions

**John J. Ceremsak**, **Basil M. Kahwash**, **Holly Budnick**, **David T. Kent**, project conception, manuscript preparation, manuscript revision.

## Disclosures

### Competing interests

Kent: Invicta Medical Inc: Consultant, Research Support; Inspire Medical Systems Inc: Research Support; Nyxoah SA: Scientific Advisory Board Member, Intellectual Property Interests, Research Support. This research was supported by research funding and provision of equipment from Invicta Medical Inc.

### Funding source

None.
